# RISE: an open-source architecture for interdisciplinary and reproducible human–robot interaction research

**DOI:** 10.3389/frobt.2023.1245501

**Published:** 2023-12-06

**Authors:** André Groß, Christian Schütze, Mara Brandt, Britta Wrede, Birte Richter

**Affiliations:** ^1^ Medical Assistance Systems, Medical School OWL, Bielefeld University, Bielefeld, Germany; ^2^ Center for Cognitive Interaction Technology, CITEC, Bielefeld University, Bielefeld, Germany; ^3^ Interactive Robotics in Medicine and Care, Medical School OWL, Bielefeld University, Bielefeld, Germany

**Keywords:** human–robot dialog, HRI studies, scenario management, explainability, Wizard of Oz, autonomous HRI, framework

## Abstract

In this article, we present RISE—a **R**obotics **I**ntegration and **S**cenario-Management **E**xtensible-Architecture—for designing human–robot dialogs and conducting *Human–Robot Interaction* (HRI) studies. In current HRI research, interdisciplinarity in the creation and implementation of interaction studies is becoming increasingly important. In addition, there is a lack of reproducibility of the research results. With the presented open-source architecture, we aim to address these two topics. Therefore, we discuss the advantages and disadvantages of various existing tools from different sub-fields within robotics. Requirements for an architecture can be derived from this overview of the literature, which 1) supports interdisciplinary research, 2) allows reproducibility of the research, and 3) is accessible to other researchers in the field of HRI. With our architecture, we tackle these requirements by providing a *Graphical User Interface* which explains the robot behavior and allows introspection into the current state of the dialog. Additionally, it offers controlling possibilities to easily conduct *Wizard of Oz* studies. To achieve transparency, the dialog is modeled explicitly, and the robot behavior can be configured. Furthermore, the modular architecture offers an interface for external features and sensors and is expandable to new robots and modalities.

## 1 Introduction

**FIGURE 1:**
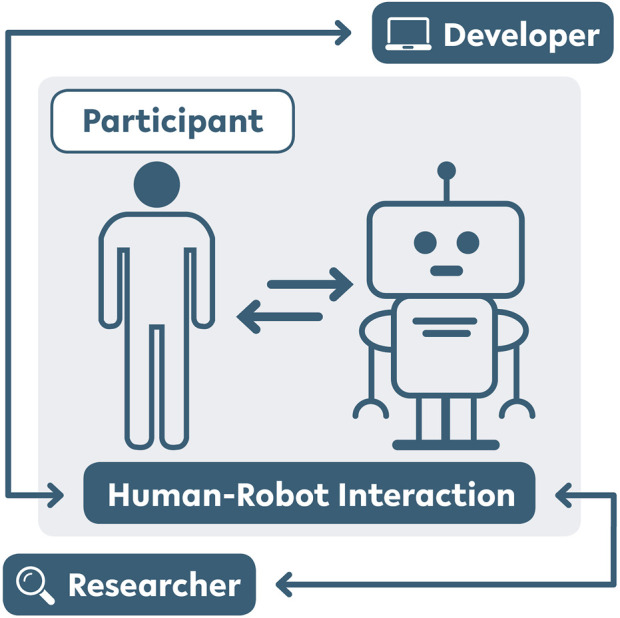
Stakeholder in *Human–Robot Interaction* studies: (1) the *participant*, (2) the *developer*, and (3) the *researcher*.

Despite the high hopes that have been put on robots as powerful resources to address societal challenges, such as care for people with special needs—especially in societies facing dramatic demographic changes—*Human–Robot Interaction* (HRI) research has still not produced helpful and acceptable assistance robots for real-life problems. In recent research, it became clear that HRI is embedded in a rich social environment that exceeds the mere dyad of the robot and interaction partner, which still is often at the heart of HRI research. Furthermore, the research of HRI itself involves more stakeholders that need to be considered, as shown in Figure 1: (1) the *participant—*the human interaction partner in the HRI; (2) the *developer* who is responsible for modeling the dialog for the HRI study; and (3) the *researcher* who is interested in exploring research questions related to HRI. It should be noted that in real-life applications, especially in the context of assistive robotics, there are even more stakeholders involved, such as care persons or family members, who need to be able to configure the robot behavior according to current needs. In the present work, we group these stakeholders—the researcher and developer—into one category, as both require an interface that allows for configuration of the interaction. However, we are aware that they have extremely diverse needs and resources and will likely require different interfaces and concepts in the future.

While previous research has mainly looked at the interaction partner, i.e., the participant in HRI studies, we focus on the developer and the researcher. Even in interdisciplinary research teams comprising computer scientists and other experts, e.g., linguists or psychologists, the interaction implementation is carried out by researchers who are experts in robotic software development. Yet, this requires intimate knowledge of the underlying concepts of linguistics, psychology, or other cultural sciences as it is the operationalization of the respective theoretical constructs.

In our transregional collaborative research center^1^ *TRR 318 Constructing Explainability* ^2^ (Rohlfing and Cimiano, 2022), we investigate explanations as a process of co-construction between humans and artificial intelligence, e.g., in the embodiment of a robot. In a collaborative, interdisciplinary approach, the mechanisms of explainability and explanations are being investigated in interdisciplinary teams with researchers from various fields, such as linguistics, psychology, sociology, and computer science. One method for investigating the process of co-constructing explanations is the application of HRI studies. By enabling other disciplines to be part of the modeling process by shaping social dialogs, we encourage collaborations. For this purpose, we provide an open-source architecture for interdisciplinary research.

We define a computational model as a model that specifies causal relationships in the processing and synthesis of interactional stimuli and behaviors in a very detailed way. According to our understanding, a computational model has equivalent characteristics to a theoretical model, which allows drawing what-if-inferences and provides explanations of the underlying processes (Ylikoski and Aydinonat, 2014). Implementing an HRI scenario in a robot is, thus, the process of composing a (specific) model of the interaction. From an epistemic perspective, it is problematic that such models are created mostly by computer scientists or roboticists with limited expertise in the theoretical linguistic and psychological foundations of human interaction.

Encouraging researchers from different disciplines to work together on developing an interactive robot system would allow the formulation of a joint theoretical model to which more than one discipline can contribute. An architecture that emerges from such a collaboration would, thus, represent a system that enables researchers to summarize their insights in a common language. With this approach, we present a further step toward more interdisciplinarity in the research on social dialogs with robots.

Another aspect that is virulent for HRI research is the lack of reproducibility. To achieve complex behavior in robots, non-trivial system architectures are required. However, social behavior is subject to a myriad of influential factors that are difficult to reproduce. In this context, reproducibility is required at different levels. *Replication—*a mere re-run of a study with only minor parameter variations—and *reproduction—*a study is carried out in a different laboratory—are necessary to ascertain reliability, while *conceptual reproducibility* aims at specifying the conditions under which a finding holds true and ascertains generalizable results (Gunes et al., 2022). To achieve conceptual reproducibility as well as replication, theoretical principles and practical resources are needed that allow us to specify what kind of variability can be neglected and which variations are relevant (Gunes et al., 2022).

A range of approaches exist that support the development of interactive robots with extension interfaces to add new functionalities through new modules and, thus, to contribute to reproducible science. Design and implementation of HRI scenarios often include recurring subtasks and patterns in diverse representations. These comprise, among other things, the processing of environmental inputs (e.g., human behavior) via sensors, execution of corresponding behaviors on (robotic) actors, and communication with different scenario-related backends, as well as the representation of interaction patterns or tasks. To this end, there have been several initiatives that aim at creating reusable robotic software, with numerous robotic systems and concepts developed in recent decades. Nesnas et al. (2003) presented CLARAty, an architecture for reusable robotic software, which was designed to reduce the implementation effort for every researcher and to simplify the integration of new technology. The architecture decouples the functional and decision layers and provides generic reusable robotic software for the locomotion and manipulation domain on various mobile platforms. CLARAty was adopted by several research institutions. However, it focuses on robot manipulation and navigation rather than interaction and has not been designed for interdisciplinary research.

Furthermore, several middleware frameworks, such as the *Robot Operating System* (ROS) (Quigley et al., 2009), *Robotics Service Bus* (RSB) (Wienke and Wrede, 2011), or YARP (Metta et al., 2006), have been proposed to manage the many challenges faced in robotics. Currently, the ROS is the “*de facto* standard for robot programming” (Estefo et al., 2019). One goal of the ROS is to enable non-roboticists to quickly create software that includes sophisticated robotic features such as path planning or grasping. The collaborative approach of the ROS can be beneficial for reproduction and replication efforts. However, the ROS requires sophisticated programming skills to develop an architecture for an interactive robot. Moreover, many current HRI experiments still cannot be reproduced easily by interested researchers to confirm the reported finding due to missing information about relevant research artifacts. It can be highly relevant what kind of speech or object recognition module has been applied to understand, e.g., the limitations of the system, or what kind of information is logged. These components are often located in different distributed repositories and use diverse build environments (CMake, Setuptools, Catkin, qiBuild, etc.). To reproduce an HRI study, all software components and their documentations must be available. Despite the current discourse on reproducibility, this rarely ever happens (Gunes et al., 2022). For reproducing whole-interaction studies, Lier et al. (2014) proposed the *Cognitive Interaction Toolkit* (CITK) for the aggregation and discovery of required research artifacts and an automated software build and deployment. The toolkit also provides an experiment description which allows repeatable execution and evaluation (Lier et al., 2017). However, while targeting reproducibility, this approach does not focus on interdisciplinary research, i.e., on enabling other disciplines to conduct their studies.

These lessons from robotics research and engineering, together with our initially stated goals of interdisciplinarity and reproducibility, can be summarized in the three main requirements for an architecture for HRI research: 1) **interdisciplinarity**: to support interdisciplinary research, the robot behavior needs to be *configurable* through an intuitive interface which is often realized via a *visual control interface*. Moreover, this requires that the internal processes are made *transparent through visual monitoring* to allow monitoring and correction. It should be noted that both interfaces can also be provided through text, which obviously would be less intuitive; 2) **reproducibility**: to enable modular exchanges of the robot functionalities, *extension interfaces* that allow us to integrate existing and evaluated modules are important. While modularity as a key feature for reproducibility is a measure of quality in a system’s engineering, frameworks and systems are often tailored to specific hardware platforms, and the middleware often restricts the availability of existing software modules. The ROS as a *middleware* with a large research community has emerged over the last few years. Varied archives of ROS-compatible modules have been developed. Regarding *platform independence*, some systems are proprietary and only work with limited hardware. Finally, for reproducibility, the system needs to allow not only for *Wizard of Oz* (WoZ) studies but also for an autonomous control mode, which is necessary for the final evaluation steps; 3) **accessibility**: importantly, systems need to be available, which requires not only the *source code* and it being *installable* with current libraries but also a systematic *documentation* allowing us to understand the functionalities of the investigated modules and to modify them for our own research purposes. Finally, the *licenses* should support public availability and use to allow for free distribution in the research community. In the following, we discuss existing frameworks for dialog and behavior modeling on robots regarding these requirements to support interdisciplinary research and reproducibility in HRI research.

## 2 State of the art in modeling a social dialog with robots

Social dialogs can be modeled using various tools from different sub-fields in robotics. In the following, we discuss tools from the field of dialog management and the advantages of specific *Software Development Kits* (SDKs) for popular robots or platform-independent frameworks for designing multimodal HRI.

### 2.1 Spoken dialog management in HRI

For implementing task-based dialogs based on speech, a wide range of research methods for dialog management exist, but only few exhibit the desired features and are still available for current research. A recent trend in research is the usage of reinforcement learning (Zhao et al., 2019). Bayesian networks or *Partially Observable Markov Decision Processes* (POMDPs) became key technologies of dialog management. These approaches train statistical models mostly based on dialog acts from training data in the domain that can then predict the next optimal dialog act given a current world state, i.e., mostly, the verbal or textual input of a user and some information about the dialog history, the interaction goal, or the environment. In these approaches, the complexity of the interaction situation is reduced to a world state that represents this information in a very discrete way. As the number of states in such a system strongly influences the search space, i.e., its computational costs, these systems are limited by dramatically reducing the information stored in these states. Although these approaches can improve the task success rate of end-to-end task-based dialog systems in a predefined scenario, they are of little use for conducting HRI studies. As prior training data are needed, domain portability is hardly possible, and the probability parameters need to be learned (or handcrafted), which tends to be expensive for more complex interactions due to a lack of datasets for task-oriented dialogs. Furthermore, the robot behavior may not appear deterministic from a user perspective as small changes, e.g., in the world state, may lead to large changes in the robot behavior. Moreover, the behavior of the robot becomes difficult to track as many variables are only implicitly considered. All this hampers the interpretation of HRI results. More traditional research methods for dialog management are *Finite-State Machines* (FSMs) (Hori et al., 2008; Ren et al., 2015; Yi and Jung, 2017). However, in less restricted interactions, which introduce more states, the dialog graph must be enriched. This leads to a fast-growing population of states which become difficult to handle.

Peltason and Wrede (2010a) proposed to model dialog for HRI based on generic interaction patterns. An interaction pattern describes recurring and configurable dialog structures on a general level and can be formalized as a transducer augmented with internal state action. Their toolkit PaMini provides a generalized description of the interaction structure, which is multimodal, mixed-initiative, and scenario- and robot-independent, and, thereby, supports rapid prototyping of dialog flows in *State Chart eXtensible Markup Language* (SCXML). Thus, it counteracts the lack of generalizability of previous HRI dialog systems (Peltason and Wrede, 2010b). However, it misses introspection capabilities into the current state of the interaction. The researcher cannot easily monitor the behavior of a robot or intervene in the behavior and control the dialog flow. Furthermore, the source code of the toolkit and the documentation are no longer freely accessible but only privately installable^3^.

IrisTK, developed by Skantze and Al Moubayed (2012), is a toolkit for the rapid development of real-time systems for a multi-party face-to-face interaction for the robot head Furhat. Like PaMini, it provides a set of modules for multimodal input and output, and the dialog flow is also modeled with state charts in SCXML. In contrast to several other toolkits, the source code of the system is still available, but unfortunately, the linked documentation^4^ is no longer accessible. In addition, monitoring (or changing) the current dialog state is not easily possible.

The incremental processing toolkit, InproTK, is an extensible architecture for incremental processing, with a focus on components for incremental speech recognition and synthesis (Baumann and Schlangen, 2012). A combination of the two frameworks, PaMini and InproTK, has been implemented by Carlmeyer et al. (2014). Although the source code is still available, the main developer recommends discontinuing InproTK^5^.

Lison and Kennington (2016) presented a hybrid approach, where the dialog state is represented as a Bayesian network. Their toolkit, OpenDial, for modeling spoken dialogs relies on the information state approach (Traum and Larsson, 2003). The domain models are specified using probabilistic rules, where unknown parameters can be estimated. The source code is still available, but the main website with the documentation^6^ is not reachable. In contrast to other toolkits, OpenDial provides some visualizations about the current dialog state.

The presented toolkits for modeling dialogs offer suitable options for designing HRI studies. However, they are only moderately usable for non-computer scientists due to missing capabilities to introspect the current state of the interaction. In addition, several toolkits are not available anymore, or their documentation is missing. However, the presented frameworks are based on good HRI modeling techniques, which are discussed further in Section 3. Most of the toolkits do not provide introspection capabilities, such as visualization, to monitor the current dialog state.

### 2.2 Behavior control for HRI studies

Some frameworks address different stakeholders and could be used more interdisciplinarily. In the context of social robotics, Siepmann and Wachsmuth (2011) developed a modeling framework for reusable social robotic behavior. They define behavior patterns (skills) to construct complex social behavior based on *sensors* and *actuators* as building blocks for social robot behavior. These sensors and actors could be a simple abstraction of a real hardware sensor or a more complex abstraction based on different software components. This framework allows for the reuse of skills, even for less experienced computer scientists. In addition, it makes the task analysis between different platforms more comparable. Their implementation Bonsai^7^ is still available. However, introspection capabilities into the current state of the interaction are not provided.

Glas et al. (2012) also addressed different stakeholders: programmers and interaction designers. Their framework enables the development of social robotics applications by cross-disciplinary teams by combining a modular back-end software architecture with an easy-to-use graphical interface for developing interaction sequences. They present a four-layer robot control architecture: (1) the robot-driven layer, (2) the information processing layer, (3) the behavior layer, and (4) the application layer. Unfortunately, the source code of their framework (Interaction Composer) is no longer accessible.

For some robots, special SDKs are developed; for example, the robots Pepper and NAO can be programmed using the NAOqi Framework. In addition, the application Choregraphe (Pot et al., 2009) offers the possibility to (1) create animations and behaviors, (2) test them on a simulated robot, or directly on a real one, and (3) monitor both robots. Choregraphe uses a visual programming technique, which allows non-experts to create their own scenarios, which can encourage interdisciplinary research. However, even if Choregraphe is the standard for programming interactions with Pepper or NAO, the replication of such scenarios with other robots is not easily possible because other robot platforms are not supported and cannot be integrated. In addition, own processing modules cannot be easily integrated. Choregraphe^8^ is well documented, and the binaries are downloadable, but the source code is not available, which makes it difficult to extend.

In the ecosystem of the ROS, Lu and Smart (2011) presented a robot control interface (Polonius). It should allow non-programmers to run WoZ-style experiments. Unfortunately, the documentation has been “under construction” for several years^9^. Furthermore, Rietz et al. (2021) developed an open-source WoZ interface for the robot Pepper (WoZ4U). It allows non-experts to conduct WoZ interaction studies. A configuration file, which saves experiment-specific parameters, enables a quick setup for reproducible and repeatable studies. The tool and its documentation are still available^10^. However, the interface is limited to the robot Pepper, and no introspection into the inner processing of robot behaviors is available. The lack of connection to the ROS makes it difficult to expand it to other modules or platforms.

Table 1 summarizes the presented tools regarding their accessibility, support for interdisciplinarity, and reproducibility in HRI research.

**TABLE 1:** Overview of different tools regarding their accessibility, support for interdisciplinarity, and reproducibility in HRI research.

			Accessibility			
	Interdisciplinarity	Reproducibility	Source code	Documentation	License	Installability
PaMini	Models dialog through configuration of interaction patterns with XML	Generic interaction patterns, scenario- and platform-independent	✗	✗	**?**	Library can be downloaded from the mvn repository
IrisTK	XML-based authoring language for dialog flow control	Supported robot is Furhat, scenario-independent	✓	✗	GNU GPL	“iristk install” is only supported on Windows
InproTK	Implements part of the general abstract model of incremental dialog processing	Concept of IU model allows modular design, scenario- and platform-independent	✓	✓	Free BSD	Installable via Gradle
OpenDial	Models dialog through probabilistic rules (XML), visualization of the dialog state	Probabilistic rules can be scenario-independent, platform-independent	✓	✗	Free BSD	Installable via Gradle
Interaction Composer	For programmers and interaction designers through visual programming	Modular through four-layer robot control	✗	✗	**?**	**?**
BonSAI	Addresses less experienced computer scientists (sensor and actor concept)	Re-use of skills (scenario-independent), platform-independent	✓	✓	GNU LGPL	Installable via Ant/Maven
Polonius	Provides WoZ GUI (can be configured via YAML)	Platform-independent through ROS actions	**?**	✗	**?**	No information available on how to install it
WoZ4U	Provides WoZ GUI	Only for the Pepper robot	✓	✓	MIT	Installable via a Docker container
Choregraphe	Interaction design by visual programming, visualization for monitoring and controlling	Supported robots are Pepper and NAO	✗	✓	**?**	Binary download available

## 3 Design and conceptualization

The review of existing tools and frameworks has shown that there are many good approaches, but they are not suitable for the desired purposes. As discussed in Section 1, accessibility and usability are imperative for use by other researchers. To increase usability, the software program should be easily accessible and well documented. Furthermore, the software license should allow general use and the (source code) expansion with other robot platforms or processing modules. To be able to connect these easily, a middleware standard in robotics, e.g., the ROS, should be used. In the following, we derive requirements for an architecture which could support **interdisciplinary** research and allow designing **reproducible** HRI research.

**Configurability**: To enable researchers to develop customizable and personalized robotic studies, a fully configurable system needs to be designed. To reach this level of customization, the fundamental information structures should be easily configurable. Therefore, an explicit formalization of the human–robot communication is needed. Three communication structures for the explicit modeling of human–robot dialogs have been identified. (1) *Dialog act*: A robot can communicate with humans in terms of generating actions as output. In most frameworks, e.g., in PaMini (Peltason and Wrede, 2010b) or OpenDial (Lison and Kennington, 2016), the concept of a dialog act is implemented. A dialog act represents the meaning of an utterance; it has a certain communicative function and a semantic content (Austin, 1975). In classical language processing tools, this output is mostly speech based (Stolcke et al., 2000; Ahmadvand et al., 2019). However, interaction is multimodal, and thus, these actions can be realized by verbal and non-verbal (e.g., gestures) communication channels. This is crucial for the robot’s perceived social intelligence, its ability to communicate, and the extent to which the robot can be trusted (Tatarian et al., 2022). Fernández-Rodicio et al. (2020) proposed the *Communicative Act* (CA), an individual composition of such communication modalities that can be described as the atomic parts of a robot’s behavior. The CA can be parametrized and combined in a hierarchical manner to fulfill the needs of the robot applications. This combination of basic CAs results in more complex, reusable blocks (*Complex Communicative Act* (CCA)). Such sequences of consecutive actions must be configurable in order to enable the expression of individual robot behavior in the scenario. In addition to the static behavior exhibited by a robot, the interaction between humans and robots is essential to achieve a complete dialog. This interaction is described by signals on behalf of the humans in the dialog. Examples of such interactive features include voice inputs or other sensory elements such as attentional parameters or emotions. (2) *Dialog policy*: The interplay of CAs between the human and the robot is the dialog discourse. To decide how to react to an act, a dialog policy is needed (Greaves et al., 2000). As discussed in Section 2.1, different methods for the design of such policies exist. However, the robot’s reactions to the human’s CA should be configurable as an interaction rule (IR). These could be generic, such as in, e.g., PaMini (Peltason and Wrede, 2010b), or reusable, such as in OpenDial (Lison and Kennington, 2016). (3) *Dialog context*: To enrich a dialog with information from the environment or context, an additional structure is needed that can share information between the human and robot. This type of memory is required to make information from the dialog history accessible to the robot. It is, therefore, important to make this shared memory area accessible to the robot and to other components in the system. This flexibility allows for rapid development, scalability, efficient experimentation, and development time as well as effort reduction. By enabling the easy and intuitive configurability of these fundamental interaction structures in a dialog in a clear and accessible format, collaborative work on HRI studies within interdisciplinary teams is simplified and more accessible.

To address the issue of repetitive tasks and alleviate the increased workload associated with reprogramming HRI studies from scratch, it is necessary to establish a generalized definition for the study. The interaction and behaviors for a dialog in HRI should be defined as high-level actions to increase the understandability, extensibility, and reusability for experts as well as non-experts. By defining these actions at a higher level, it becomes effortless to configure and reuse them. By enabling reusability beyond specific scenarios, the need for designing new behaviors repeatedly is reduced. This results in the creation of a set of usable behaviors.

**Introspection and robot control**: A crucial objective of a system designed to control robots within interdisciplinary teams is to facilitate collaboration and comprehension of HRI scenarios for both experts and non-experts. This requires a system that can represent the inner states of a robot and its corresponding behavior in a comprehensible way. Approaches for such visualizations are, e.g., provided by Lison and Kennington (2016) and Pot et al. (2009), which provide a visual insight into the current dialog state. Insights into the inner processing states of robot behaviors provide valuable in-depth analysis capabilities for computer scientists. It allows experts to monitor and understand how the robot behaviors are generated, to debug issues, and to improve the overall performance and reliability of the system. The given transparency of a system empowers experts to make informed decisions and enhance the efficiency of the HRI scenario. For non-computer scientists, transparency in robot behavior fosters understanding and trust in the system. Insights into the inner processing states in a visual and intuitive manner lead to more trust and understanding of the system in general (Papenmeier et al., 2022). This promotes user confidence and acceptance, enabling non-experts to better grasp the capabilities, limitations, and intentions of the robot (Hindemith et al., 2021). In an interdisciplinary team, it also encourages collaboration and effective communication between non-experts and robotics experts. A system that is comprehensible for experts on all levels with a shared visualization tool also enables a high level of exchange about the system’s behavior. Furthermore, intervening with the behavior and controlling the dialog are needed to conduct WoZ interaction studies, such as the visualizations of Lu and Smart (2011) and Rietz et al. (2021).

**Extensibility and robot independence**: One goal of a reproducible architecture in a system for interdisciplinary research is to enable the reproduction, replication, or re-creation of previous HRI work (Gunes et al., 2022). Furthermore, it should reduce the effort of developing new HRI studies. This can be done by enhancing the reusability and extensibility. To achieve an extensible and reusable system, the conceptual idea is to develop discrete high-level components. To facilitate system extensions, the implementation of an interface is another crucial element of a reproducible architecture. A uniform, easy-to-use interface allows for the use of additional features as external components in the architecture. The interface allows for the integration of expert features into the system, enabling the reuse of systems within the architecture and alignment with the concept of distributed systems (Tanenbaum and van Steen, 2007). The ability to exchange such features with other scientists or project members fosters interdisciplinary collaboration and enhances the overall quality of the work. Furthermore, to ensure the continuous utilization of these reusable components, the application should be independent of any specific robot. This flexibility enables switching to a different robot during development without requiring additional effort. It allows a collaboration team to reuse all developed components and studies when deploying the application to a new robot. In addition, replication of studies with different platforms is a key aspect of conceptual reproducibility.

Additionally, when a team has created a collection of functionalities for their primary robot, switching to a new robot would typically require modifying all communication with the backend of the robot—provided that the same modalities and programming language are utilized. To address this extended workflow, it is necessary to define the potential robot functions directly within the system and to subsequently translate them for the specific robot. This approach enables switching between robots if the target robot is already integrated into the system, allowing for immediate compatibility.

**System requirements:** To sum up, in this work, we present an architecture that enables the development, implementation, and observation of HRI scenarios and simplifies the work on this topic for interdisciplinary teams. Built on the core concept—the developed system—which was designed to facilitate interdisciplinary research and to ensure a reproducible architecture, it can be characterized by the following requirements:

1. **Configurability** of robot behavior, interaction patterns, and environment information.
2. **Introspection** by a user interface to explain robot behavior.
3. **Robot control** allows autonomy and fully controlled execution of robot behaviors.
4. **Extensibility** of the programming interface for using external features and sensors.
5. Addressing **robot independence**, which allows the use of new robots and modalities.

## 4 RISE

This article introduces **R**obotics **I**ntegration and **S**cenario-Management **E**xtensible-Architecture (RISE), an implemented architecture designed to facilitate collaborative research in HRI in interdisciplinary research teams and to support reproducibility. **RISE** ^11^ **is an open-source software application**, published under an MIT license, developed by computer scientists to efficiently develop HRI scenarios in collaborative teams. **The documentation of RISE is also published online** ^12^. The system itself is mainly developed in Unity3D^13^ and offers a variety of options for expandability. The following section introduces the main architecture (Section 4.1) of the system, which aims to match the requirements (Section 3) in collaborative works. By introducing the fundamental components of the architecture (Section 4.2), this paper elucidates the mapping of human–robot dialogs in the field of HRI. To enable interdisciplinary teams to exchange information about HRI scenarios, the introspection capabilities of the system are supported by a *Graphical User Interface* (GUI) (Section 4.4).

### 4.1 Architecture

**FIGURE 2:**
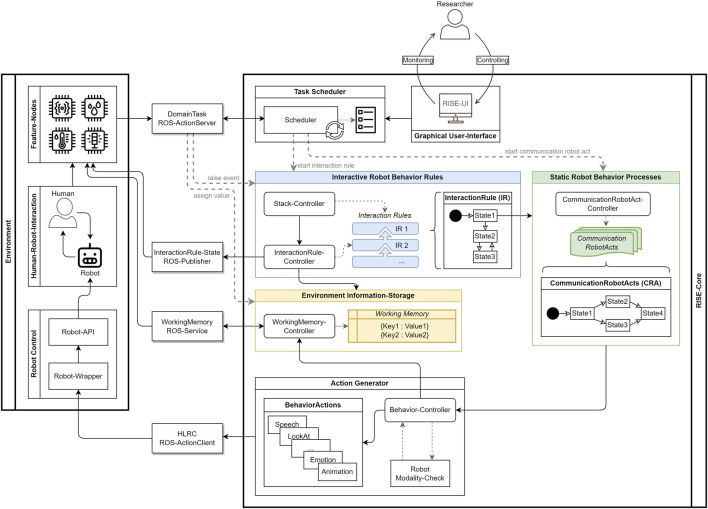
RISE as a three-level architecture, with the components’ environment, the RISE-Core as the system itself, and the ROS interface in a setup of an HRI scenario.

RISE is designed to facilitate the development of HRI scenarios for individuals with varying levels of expertise, including both experts and non-experts. One application of RISE is serving as a control center for the automated or manual control of a robot during dialogs. Therefore, the system is described as a three-level architecture (Figure 2).

#### 4.1.1 Environment

In every HRI scenario, there is a distinct context in which interactions between the robot and human occur. This part of the world is called the *Environment,* as shown in Figure 2. In our visualization, the environment does not only encompass the *Human–Robot Interaction* communication but also includes components which are responsible for the direct execution of the robot’s behavior (*Robot Control*). Moreover, it involves additional components that process external signals from the environment as inputs (e.g., the recognition of a human *Communicative Act*). These standalone components, named *Feature-Nodes*, process information from the communication between the human and robot. Here, the emphasis lies on the interaction between robots and an individual human. Dealing with multiple individuals would require additional processing components. Information processing in this context can encompass various aspects such as language processing, emotion recognition, and attention recognition.

#### 4.1.2 RISE-Core

The RISE-Core (the RISE system itself) is the central communication interface between researchers and the robot. The system manages the basic structures of communication in a dialog between the human and robot. These include *Static Robot Behaviors* in terms of **Communication Robot Acts** (CRAs) (Section 4.2.1), *Interactive Robot Behavior Rules* as **Interaction Rules** (IRs) (Section 4.2.2), and an *Environment Information Storage* as a **Working Memory** (WM) (Section 4.2.3). The organization, processing, and execution of these structures in an HRI scenario are some of the main system tasks. These system tasks include, among other things, the storage of the structures to be parsed from configurations in plain text and reception, as well as provision of various interfaces to control the execution of the configurations. A *Task Scheduler* is responsible for controlling incoming *domain tasks*, which can trigger the initiation or stopping of behaviors in RISE. These tasks are represented by ROS *Action Goals* ^14^ and can either be sent to RISE from external components via an *Application Programming Interface* (API) or executed directly via the GUI. The user interface also offers the option of monitoring robot behavior according to the internal robot states in a current scenario. From accepting *Domain Tasks* to executing them, a task goes through various states and is managed by the scheduler and a priority stack. The *Stack Controller* maintains the list of all running IRs and executes them in the order of priority. Execution of robot behavior based on a CRA is up to the *Action Generator*. The action generator produces robotic multimodal behaviors as output for a scenario in the environment. For this purpose, an internal *Behavior Controller* executes the CRA state machine-like (Section 4.2.1). Finally, RISE can store information about the environment via the WM, which is accessible for internal and external processing.

#### 4.1.3 ROS interface

The third layer in the architecture is the ROS interface between the environment and RISE. The complete communication with RISE itself is realized via the ROS. ROS *Action Servers* are used in RISE to receive tasks from external components or from initiations via the GUI. The generated output in the form of robot behaviors is also sent from RISE to the environment via the ROS. For this purpose, an ROS *Action Client* was implemented for every possible action. This type of actions also allows RISE to receive feedback from the robot about action processing states. Based on the *HLRC* (Schulz et al., 2016) server message structures, these messages are received by a *Robot Wrapper* and transformed into robot commands through *Robot-APIs*. RISE supports two additional ROS interfaces to obtain information about the internal processes within the system. The IR *State-Publisher* enables external components to receive real-time updates on state changes in IRs. Furthermore, RISE provides the capability to request stored information from the WM via a **Working Memory Service**.

### 4.2 Communication structures

The main communication structures represent the basic building blocks, which constitute the smallest but also the most important components within the architecture. These structures correspond to the specific elements within individual components that compose segments of dialogs, ranging from simple static robot behaviors in *eXtensible Markup Language* (XML) (Consortium, 1994)-based configurations (Section 4.2.1) to interactive behavior patterns that can be described by SCXML (Barnett et al., 2007). Finally, a shared memory (Section 4.2.3) is introduced for these two structures, which makes information about the environment accessible in the *JavaScript Object Notation* (JSON) (Pezoa et al., 2016) format.

#### 4.2.1 Communication robot act (CRA)

RISE aims to facilitate communication between robots and humans across both verbal and non-verbal communication channels. To achieve this goal, the robot’s contributions to the dialog are broken down into atomic actions. These actions should easily be describable in text form and have the versatility to be applied uniformly across various robot platforms. Configurations of actions in this article follow the *Behavior Markup Language* (BML) (Kopp et al., 2006) approach of an XML-based annotation for designing multimodal behaviors for robots. Like previous work (Groß et al., 2022), the focus is mainly on behaviors for communication in a dialog but is not limited to these actions.

• **Speech**: Processing the verbal voice output of a message.
• **LookAt**: Looking at certain targets in world space coordinates.
• **Emotion**: Executing predefined emotional states (e.g., happy, sad, and fear).
• **Animation**: Executing predefined animations (e.g., head node and shake).

**FIGURE 3:**
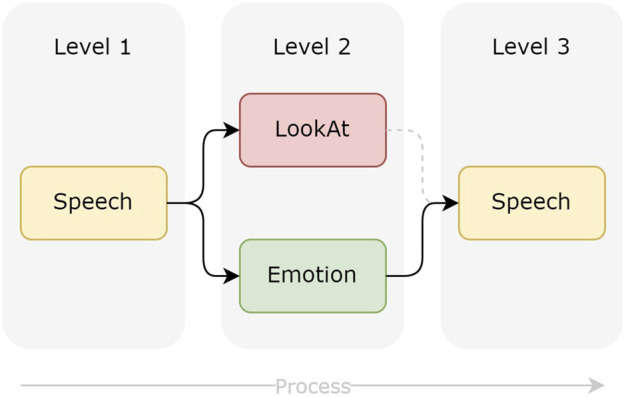
Conceptual visualization of a CRA with four actions on three execution levels.

By combining these actions consecutively, it becomes possible to express sequences of behavior. A short sequence, which describes a robot greeting a person, is shown in Figure 3. The robot output consists of four actions to be executed. The execution of such a sequence shows multimodal behaviors. On the second level of execution, actions are executed parallelly. The first *Speech* action starts the sequence, and after the ending of this action, the actions *LookAt* and *Emotion* start simultaneously. The last *Speech* action ends the overall sequence and starts by the end of *Emotion*. A hierarchy of behaviors is created by parallel and in series-connected actions.

The sequence of actions shown in Figure 3 can be structured and parameterized using XML configurations (Listing 1). These configurations are interpreted by the system and transmitted to the robot, enabling it to execute the actions in a meaningful manner. Each action is represented by an overall action tag and its corresponding parameters. A *Speech* action, e.g., requires a text message for the robot’s speech synthesis. The action *LookAt* requires different world coordinates to allow the robot to look at a target. *Emotion* is described by a predefined set of values: value *1* represents the emotion *Happy* in this example. By introducing the *waitForActions* parameter, it becomes possible to configure waiting conditions for an action.

Listing 1 XML Configuration for CRA - Greeting.

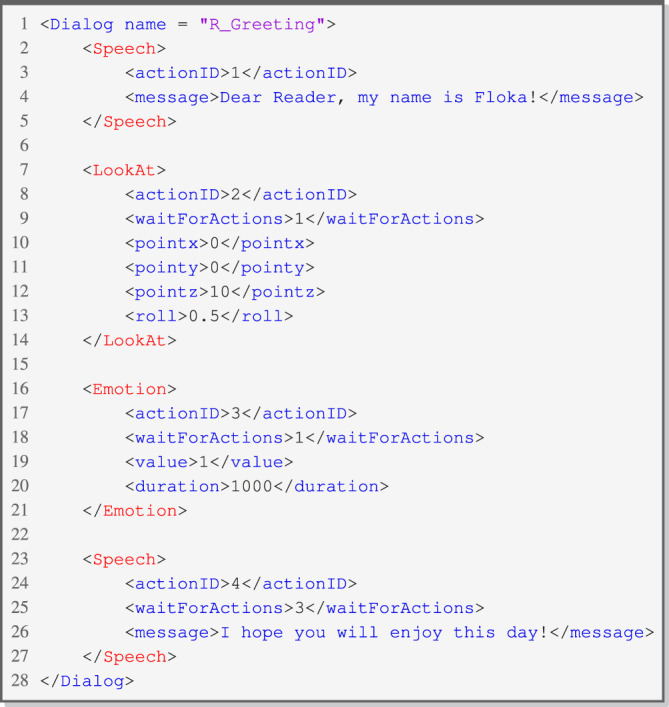

#### 4.2.2 Interaction rule (IR)

**FIGURE 4:**
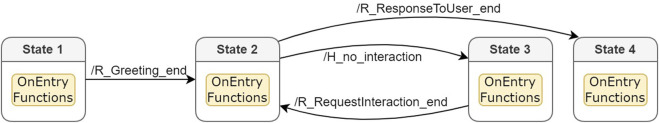
Conceptual visualization of an IR with four states.

In addition to defining static robot behavior, there is a need for a concept to articulate reactive behavior and manage contextual changes. An IR, grounded in the concept of state machines, comprises states, transitions, and functions. This concept was extended with the idea of conditional behavior when entering a state, depending on values in the memory. Figure 4 displays a rudimentary visualization of an IR with states *1–4*.

Each state includes functions and transitions. The functions will start once a state is entered (*onEntry*). The following functions are currently implemented by default.

• **start Communication Robot Act**: Starting a CRA.
• **start Interaction Rule**: Starting an IR.
• **raise Event Topic**: Raising an internal event on a specific topic with the defined value as a message.
• **assign Value**: Assigning a value to the WM.

A state waits for a corresponding event message to make a transition to another state in the state machine. These events can be raised inside an IR by calling the corresponding function (*raiseEventTopic*) with a value for the event. An event can also be raised externally by sending a *Domain Task* with the function to raise an event topic. In addition to that, every CRA automatically raises an event (*NameOfTheCRA_end*) when it has ended. Due to the state machine, IRs are described in SCXML (e.g., Listing 2). This SCXML presents a small example of an IR waiting for a specific WM value to start the interaction. While waiting for the value to reach the desired threshold, the IR cycles between *State 2* and *State 3*. To augment these capabilities, it is feasible to execute specified functions conditionally by establishing dependencies on a memory. The operators and types for these expressions are implemented based on a *Logic Expression Parser* ^15^. They include logical expressions (or, xor, and, = =, ! =, not, <,> ), arithmetical operators (+, -, *, /, $\hat{\;}$ ), and string operators for comparison (starts with, ends with, contains, = =, ! =). The results of these operations can be nested, which can lead to even more complex conditions.

RISE can manage multiple active IRs simultaneously. This leads to a conflict regarding the expected action in the case where transitions of two different IRs are waiting for the same event value. To avoid conflicts, management is carried out by the *Stack-Controller*. Each IR has a certain priority, and the one with the highest priority is assigned the value to make the transition. Moreover, when a transition is used, this IR is the most relevant at the time and, therefore, is set to the highest priority.

Listing 2: XML Configuration for IR - Repeated Interaction.

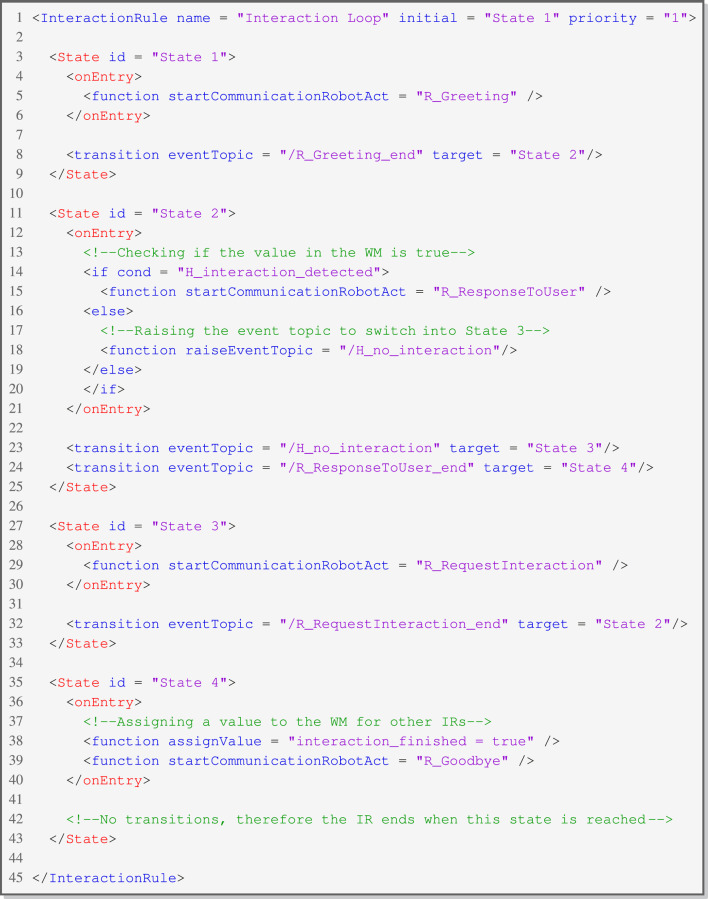

#### 4.2.3 Working memory (WM)

The WM retains previously defined environmental information as well as data generated during the HRI scenario. This kind of information is defined as JSON (Pezoa et al., 2016) key-value pairs. Listing 3 represents a small example of predefined information in the WM in the JSON format, which is accessible at system startup.

Listing 3 JSON Configuration of Working Memory.

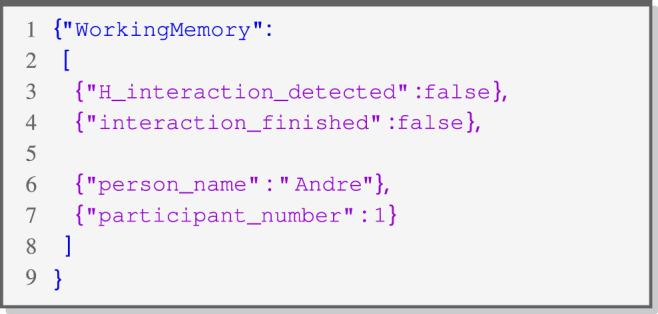

This memory can be directly used within functions in the IRs to parametrize if-else statements within conditions in a state (*onEntry*). Additionally, CRAs can use variables from the memory to load values by naming the key within an attribute configuration. The WM is also readable by external components in the architecture via an *ROS-Service*. In order to allow setting entries in the memory from outside of RISE, *Domain Tasks* can insert values into the memory with a key by using the corresponding function (*assignValue*). This type of function is handled by the scheduler but is processed directly and not scheduled on an internal list of tasks. In addition to that, the memory can also be fully controlled inside the GUI. This capability can be leveraged to establish connections with environmental information and to enhance the dialog by incorporating contextual details, such as the participant’s name. The WM in RISE is not primarily intended for the visualization of sensor data. Instead, it is utilized to grant access to information related to the logic within the defined HRI scenario. To achieve this, one approach involves explaining the decisions made within the RISE-designed scenario and making these logic blocks accessible via their return values. This presentation typically takes the form of plain text. The main emphasis here is on ensuring that the higher-level scenario remains easily understandable. In case there is a need to visualize the traceability of feature nodes that operate on raw sensor data, tools, like RViz^16^, are still viable options for this kind of data beside RISE.

### 4.3 Designing an HRI scenario

A typical interaction that can be readily modeled in RISE is a human-initiated greeting. It involves a robot detecting and acknowledging the user’s presence. This interaction is facilitated by input sensors, such as a camera, to track the user’s position, and a microphone, to capture their speech. The dialog sequence is straightforward: as the user approaches, the robot should signal its responsiveness by directing its gaze toward the user. If the user initiates with a greeting like “Hello” (or any other similar greeting), the robot should respond accordingly and greet back.

The CRAs utilized in RISE for this scenario encompass various descriptions of the robot’s greeting behaviors and the actions associated with tracking the human’s position through the movement of the robot head.

To monitor the person’s position and capture their verbal communication, RISE records these preprocessed sensory data obtained from a feature node in its WM. The dialog act detection is based on simple key word spotting in this case, but more sophisticated dialog act detection could be easily integrated. The objective is to identify whether a human is in close proximity to the robot and if the human is initiating a greeting (in this case, through speech, but, of course, a multimodal greeting detection would also be feasible). By leveraging this external sensory input within RISE, it becomes possible to create an IR that empowers the robot to reciprocate the greeting.

 Therefore, the IR needs to track the state of the human in its states and variables. The example is segmented into the following phases: is the human within close proximity, have they greeted the robot, and has the robot already greeted this person in return. When the human is close enough, their location is employed to maintain the robot’s gaze directed toward them. If they greet the robot, the robot responds with a greeting selected from various possible formulations and then proceeds to introduce itself. To avoid repetitive greetings, if an individual has been greeted, this information will be temporarily stored in the WM. Should the person move out of the robot’s field of vision, the robot head posture is reset to its default position. The technical setup for these brief example configurations is accessible online^17^.

### 4.4 Introspection: graphical user interface

**FIGURE 5:**
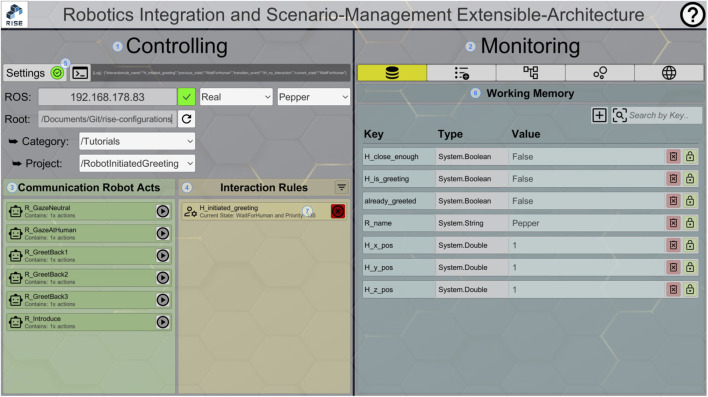
RISE GUI with loaded CRAs (3) and IRs (4) on the controlling (1) side; monitoring (2) area with the selected WM (6).

RISE not only brings the CRAs, IRs, and WM together but also offers an extensive GUI. The design of this GUI is divided into two main areas—controlling (1) and monitoring (2)—and represents the current states of robot behaviors and the overall structures (Figure 5). First, in the controlling area (1) on the left side of the interface, researchers can see an overview of all available CRAs (3) and IRs (4). The GUI enables the researcher to initiate and terminate both structures within that overview list. By visualizing the processing states, it is possible to see which of them are currently active. In addition to that, it is possible to customize different *Settings* (5) for one’s own setup. The IP address references a connection to a robot backend. Next to the ROS connection, the GUI offers the parametrization of the paths to the configuration files for the main structures and allows switching the target robot. For debugging, the execution mode of the system can be changed from *real* to *debug*. In the *debug* mode, the actions are simulated in the state machine visualization and are not directly executed and transferred to the robot.

Second, in the monitoring area (2), the interface enhances the usability of the system by providing introspection features for researchers. This is organized by a tab system with five different views. The visible tab (6) on the right side in Figure 5 represents the current memory data. Researchers can directly change values and delete or even create new entries. When changing the value, the data type updates automatically to enhance understanding if the entered value matches the expected type.

**FIGURE 6:**
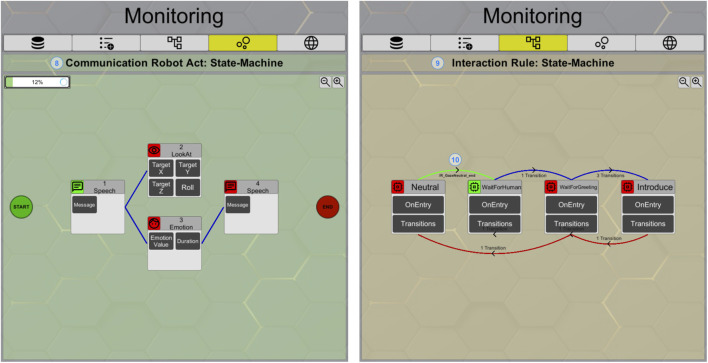
RISE GUI monitoring pages for CRAs (8) and IRs (9).

The application features dedicated monitoring pages for the CRAs (8) and IRs (9) (Figure 6). By marking an IR (7) and switching to the dedicated tab (9), the details of this IR will be displayed. The current active state and last transition used are highlighted (10). Hovering over a state reveals all defined conditions and functions for that state. The presentation for the CRAs (8) is similar. The defined actions as well as the one currently being executed can be inspected. Hovering over the visualized state gives more in-depth insights into the parameters of the action, e.g., the message inside a speech action.

**FIGURE 7:**
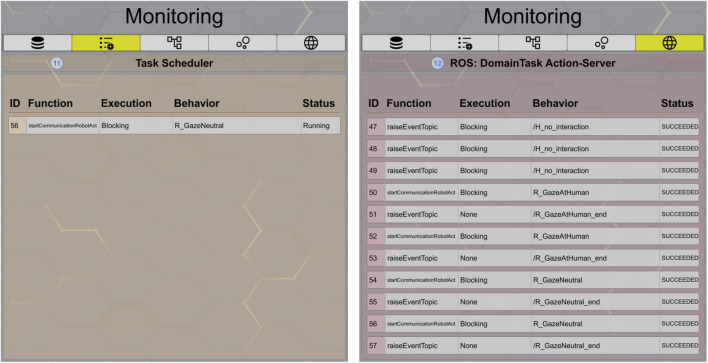
RISE GUI monitoring pages for the *task scheduler* (11) and the *domain task action server* (12).

On the left side of Figure 7, the monitoring tab of the *Task Scheduler* (11) is shown, providing an overview of tasks that are currently running or waiting. To avoid conflicts, such as two CRAs trying to use the speech action at the same time, the CRAs are designed to be executed sequentially, one after another. Therefore, the execution type is *blocking* additional CRAs if another CRA is already running. This then leads to a waiting status. Meanwhile, the scheduler displays upcoming robot behaviors. The last tab on the right side (12) in Figure 7 is dedicated to debugging and the in-depth understanding of the system. All actions, regardless of whether they were executed by an IR or a feature node, are forced to send a *Domain Task* to the *Action Server*. These incoming tasks are presented together with their corresponding function, execution type, behavior, and status in this tab for further understanding, introspection, and recording capabilities.

### 4.5 Implementation summary

We presented a system that supports interdisciplinary research and ensures a reproducible architecture. Based on the underlying concept, we outlined the key requirements (Section 3) that make collaborations within the field or HRI easier for research teams. In summary, the requirements for such a system were fulfilled as follows. **Configurability**: The configurability of robotic behaviors in terms of statically generated output and reactive rule-based behaviors is realized by implementing CRAs and IRs. To store context information from the environment, a WM offers external components, while RISE itself saves and loads information into a key value-based dictionary (Section 4.2). These tree structures can be preconfigured in XML/JSON format. They present the configurations of RISE about all possible robot behaviors and default environment information. **Introspection**: A GUI was developed to create a productive discourse within an interdisciplinary team. This allows the team to discuss internal states of robot behaviors and improve the quality of designing HRI scenarios (Section 4.4). **Robot Control**: By using IRs and the GUI, RISE enables researchers to create fully autonomous and controlled HRI studies. The IRs allow researchers to customize experiments by applying rule-based behaviors. RISE controls robot reactions to given inputs from the environment. The state machine-like structure of an IR allows RISE to execute an entire experiment fully automatically. In addition, the GUI of RISE also enables researchers to create WoZ studies in a fully controlled and non-automatic way. **Extensibility**: RISE (Section 4.1) is created as a three-level architecture, which describes the HRI scenario with all external components and information-producing nodes as the environment, RISE as a standalone system, and the ROS as the main interface for the information exchange between the environment and the system itself. By leveraging the capabilities of the ROS, RISE provides a versatile interface that enables the reception and publication of information between the system and the environment as well as external components. **Robot Independence**: The establishment of the fundamental structures enables the utilization of custom robots and actions. RISE builds on an existing system that constitutes its foundation (Groß et al., 2022). Within this architecture, the most elemental actions of a robot are represented as *Behavior Actions*. Implementing the abstract class in the source code allows for the straightforward addition of new actions for new robots. Furthermore, the system offers support for the integration of new robots.

## 5 RISE in application

To evaluate the effectiveness of RISE in facilitating the development and execution of HRI studies, this section focuses on showcasing the system’s proof-of-concept and first use cases. A walkthrough encompasses an overview of the core capabilities of the system within the context of a small HRI scenario—as evaluated by previous researchers and developers. These insights cover various aspects, including system accessibility and documentation. We aim to highlight that the development status has transformed from a mere concept into a practical and usable system. In addition, the conceptualized details are also demonstrated by exemplary real-world applications, providing practical illustrations of the implementation of RISE for researchers and developers. Finally, we summarize the functionalities and compare them to similar systems. Moreover, we illustrate the limitations of the architecture.

### 5.1 Proof-of-concept

In the following section, we aim to illustrate the test cycles and use cases that developers and researchers within our working group underwent to engage with RISE. With these examples, we intend to demonstrate that RISE can be effectively utilized by following the provided documentation, even without an extensive background in the system. In addition, we showcase the functionalities that can be readily implemented.

#### 5.1.1 Accessibility of the system

To demonstrate the accessibility of RISE, we had seven individual test persons install and utilize the system independently. The process involved installing and launching the system using the provided documentation^18^ on a newly set up laptop with pre-installed Ubuntu 20. After installation, the initial tasks included the configuration of personalized robot behaviors and their execution with the virtual robot Floka and the robot NAO. Floka is used as an in-house simulation of the robot head Flobi (Lütkebohle et al., 2010) for research purposes within our research group. Both the installation procedure and the execution of the tasks leading up to configuring a basic HRI scenario for robot control were completed by all participants without substantial assistance. Using ongoing enhancements to the documentation, we reached a point where external assistance was no longer necessary for system installation.

#### 5.1.2 Walkthrough of the functionalities

Table 2 outlines the primary features of RISE by categorizing them into tasks for researchers and developers. Developer tasks primarily involve deep engagement with the system’s source code and entail programmatically driven changes. Conversely, the researcher’s role aligns with the traditional role of a non-computer scientist, with no programming skills required. It is also conceivable for one person to take on both roles concurrently. When we emphasize interdisciplinary research teams, our aim is to underscore that both functions are essential, and there is no inherent exclusion of one role over the other. To further improve the functionalities of RISE, we had five test subjects try the system functions using the documentation of tutorials^19^. In the following, we provide a step-by-step demonstration—using a contemporary example—of how a group of student assistants with different programming skill levels establish their own joint HRI scenario for a laboratory opening session (Figure 9). The aim of the student group was to create a scenario in which a robot welcomes arriving guests in the laboratory, provides an introduction to the laboratory, and responds to questions or inquiries that come up.

**TABLE 2:** RISE use cases with the main functionalities in the application of HRI studies for each target group.

Function	Researcher	Developer
Defining robot behaviors	✓	✓
Integrating new robots	✗	✓
Integrating new actions	✗	✓
Integrating external nodes	✗	✓
Study execution/adaption	✓	✗
Introspection	✓	✓
RISE as a WoZ tool	✓	✗
RISE for recording	✓	✗

**Defining robot behaviors:** In this context, various tasks emerge, including the configuration of room descriptions, the storage of contextual data in the memory, and the establishment of interaction rules for welcoming guests on arrival. By blending atomic robot action sequences—represented by dialog elements—with the use of CRAs (Section 4.2.1), with rule-based interaction patterns (IRs, Section 4.2.2) and incorporating a memory system to retain contextual scenario information (WM, Section 4.2.3), the students gain the capability to craft their own custom scenario. The design process of these XML configurations, utilizing the principles of the BML (Kopp et al., 2006), has undergone an initial pilot study evaluation. This small-scale study (Groß et al., 2022) included a system-usability test with six participants (three computer and non-computer scientists each) investigating first concepts in a previous version of the RISE architecture. The results indicate that the usability of the scenario configuration was rated “good” by both groups.

**Integrating external nodes:** In the present scenario (Figure 9), student assistants were asked to generate multiple external nodes. Initially, a gaze recognition node was required to determine when the robot should welcome a visitor in the laboratory. Therefore, we developed a simple gaze recognition system that detects appropriate moments for the robot to initiate greetings with a human. This script was coded in Python and connects with RISE through ROS using *Domain Tasks*. As a result, greeting dialogs are promptly triggered as soon as the robot establishes gaze contact with a person. We also integrated *Automatic Speech Recognition* (ASR)^20^ as a *Feature-Node* in the RISE architecture. We combined ASR with the powerful large language model ChatGPT4all (Anand et al., 2023) to enable the robot to respond to incoming questions and engage in dialogs with visitors (see the online repository^21^).

**FIGURE 8:**
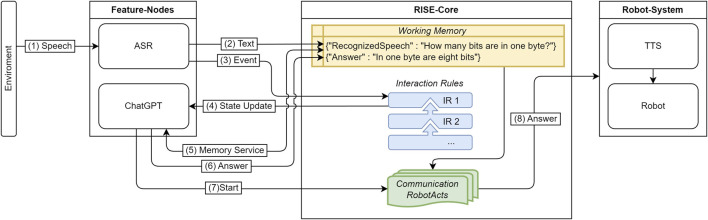
Overview of an exemplary use of external components in RISE to use ChatGPT answers in HRI.

Figure 8 presents one possibility to integrate these components in the architecture and describes the information workflow. ASR records the human speech (1) and translates it into text which is written via a *Domain Tasks* into the WM (2). Additionally, ASR sends out an event to trigger an IR (3) in order to notify a change in the WM. The change in the current state in the IR can be recognized by the subscription of the IR-state updater (4). This notification triggers the ChatGPT node to request the value for the memory key (*RecognizedSpeech* (5)). Next, the ChatGPT node generates an answer depending on the requested data and saves the information as its own entry in the WM (6). Finally, the node directly starts a CRA (7) which uses information from the WM to output a response (8) via the robot’s speech synthesis.

**FIGURE 9:**
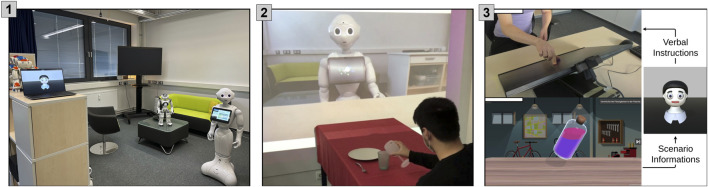
**(1)** Laboratory opening scenario. **(2)** Robot Pepper in the LabLinking scenario (Richter et al., 2023). **(3)** Robot Floka using negation in an explanation strategy scenario (Groß et al., 2023).

**Integrating new robots in RISE:** To incorporate new robots with additional actions into RISE, programming modifications need to be made directly within the RISE source code. To accomplish this, the system requires an internal representation of the robot’s name and modalities, including the set of possible executable actions. To enable RISE to execute the same configuration across different robots, it is necessary to define which actions can be executed by each specific robot. This ensures the adaptability of RISE to different robot capabilities. The laboratory opening session (Figure 9) was slated to feature only the robot Floka. However, the current version of RISE supports the robots NAO, Pepper, and Floka. No additional setup or integration of these robots is required at this stage.

**Integrating new actions in RISE:** All actions in the system are represented by the abstract class *Behavior Actions* (Groß et al., 2022). To integrate new custom actions into the system, they must be implemented and extended from the abstract class. This expansion involves various tasks, such as adding a new ROS message, implementing a function to publish the action, and extending the corresponding robot wrapper for handling the action translations to the robot. For our laboratory opening session (Figure 9), RISE lacked the inherent capability of robots to perform pointing actions. To address this in the current scenario, we introduced a *PointAt* action, allowing NAO and Pepper to gesture toward specific spatial coordinates by using their hands. The Floka robot head realizes this action by simply directing its gaze toward the designated target point in space.

**Study execution and adaption:** During a study, the main functionalities that researchers rely on are the ability to initiate and halt robot behavior as well as the capability to independently set information in the memory. These features empower researchers to have fine-grained control over the robot’s actions and the ability to manipulate the stored information within RISE. RISE can be used as a tool for WoZ or autonomous studies (see Section 5.2.1; Section 5.2.2). In the laboratory opening scenario (Figure 9), RISE does not serve as a WoZ tool. Instead, the objective is to utilize RISE as a backend application on a server enabling autonomous control of the robot.

**Introspection for monitoring:** The GUI makes it possible to view all configured structures in one interface (Figure 6). When executing robot behaviors, the states of the respective configurations in the GUI change. A fully traceable state machine with information about the current state of the robot can be viewed. Triggered rules are highlighted, and the researcher can view the inner states of each behavior and follow the state-machine flow during the execution. The introspection capabilities of the state machines for researchers were also evaluated in the first pilot study (Groß et al., 2022). Participants rated their insights into robot behavior provided by the GUI as being highly beneficial. With a qualitative examination of participant feedback, we enhanced the introspection features in the latest iteration of RISE. In our scenario (Figure 9), a potential use case involves the ability to demonstrate to guests in a different room the reasoning behind the robot’s behavior and its present state, as well as the information stored in its memory.

**RISE for recording:** Because RISE uses ROS as middleware, it is easy to record the whole study execution just by recording all ROS messages in rosbag^22^. This makes any scenario fully reproducible. All internal processing is supported by the internal logging of the system itself. A study can be completely screened from the combination of both recordings.

### 5.2 Primary applications in real-world HRI studies

In addition to the individual functions of RISE, the next goal is to show how RISE can be integrated into real HRI studies. We aim to demonstrate the versatility of RISE in various types of studies. Figure 9 shows the previously described laboratory opening scenario (1) and two additional HRI studies that were conducted across diverse application domains using RISE. Scenario (2), the LabLinking study, revolves around the real robot Pepper and employs a WoZ setup. Conversely, scenario (3), the negation study, features the virtual robot Floka and is under the autonomous control of an alpha version of RISE. Both studies were implemented by interdisciplinary teams. The two experiments tackled a diverse array of research questions. They also exemplify the extent of interdisciplinary collaboration, particularly with researchers from biology, linguistics, and psychology, to showcase the breadth of the research topics explored. For this purpose, we present the main use cases for RISE, as demonstrated in these two types of studies.

#### 5.2.1 RISE as a Wizard of Oz tool

During a LabLinking study between two laboratories at different universities, hesitation as a scaffolding strategy during distraction in HRI was investigated (Richter et al., 2023). More specifically, we were interested in the question as to whether it was possible to show that phases of distraction from and re-orientation toward the interaction could be automatically found in *Electroencephalogram* (EEG) signals. In the study, the robot Pepper (located in lab1) gave instructions to a human interaction partner (located in lab2), which provided feedback related to comprehension. Pepper explained tasks such as setting the table in an unusual way to avoid prior knowledge. Simultaneously, the robot was in a videoconference-like scenario with the participant in the second laboratory. The participant tried to follow Pepper’s instructions, when distracting background noises were played randomly. Although the participant had the impression that Pepper acted fully autonomously, its behaviors were triggered manually via a small WoZ GUI. This WoZ setup served as a first step toward a fully autonomous, distraction-sensitive assistance robot for learning new tasks based on EEG signals.

Overall, there are three options to use RISE as a WoZ tool. CRAs can be created for every possible interaction element and response required for the study design. These would then be triggered manually by the wizard while the study is conducted. Moreover, RISE also supports semi-autonomous WoZ studies. In this case, the researcher has the ability to establish IRs that can access variables within the WM. These variables can subsequently be manually populated by the wizard as part of the study. Another option is to use raised events from manually started IRs as a trigger for the main IR.

However, this study presents a setup—including four tasks—that could be reconstructed with RISE within a short time. One exemplary configuration of a solution for this setup in RISE can be found online^23^. The verbal instructions by the robot are saved in individual CRAs, which are executed by an IR. The IR is structured in a way that once Pepper has thoroughly given the instruction, the IR transitions to the next state, in which it awaits feedback from the researcher. In addition to RISE, no additional GUI is necessary here. The next instruction or command to repeat the last instruction is triggered by a manually activated IR in RISE. These IRs only raise an event value on input and immediately end after that. Therefore, the researcher is still in the position to decide what happens next and when it happens, depending on participant feedback.

#### 5.2.2 RISE as an autonomous robot controller

A study imitating a dialog between humans and robots was conducted to investigate the impact of contrastive explanations in explanatory dialogs on human understanding within task-based contexts (Groß et al., 2023). In this complex study, a team of computer scientists, linguists, and psychologists worked together to address the question as to whether instructions containing negation scaffold humans in a way that results in better action performance at the expense of longer processing time due to the linguistically demanding instructions. The research goal is to develop an assistance system that can scaffold a human by explaining task steps in an adaptive way that takes the human’s level of understanding into account.

In this study setup, the robot Floka gave verbal instructions to a human. With the help of these instructions, humans solved tasks on a touchscreen. A specially designed game on the touchscreen transmitted information about the states of both game progress and the human input back to the robot in order to enhance the dialog. The exchange of information between the robot and scenario via the ROS enabled the realization of a fully autonomous study process. To implement this autonomous approach, a state machine-like structure was embedded within the scenario itself. For the robot, an early version of RISE was embedded to receive different events from the scenario and to interact by displaying predefined robot behaviors^24^. In the present case, the experiment design was structured in such a way that the course of the experiment was managed via the touchscreen scenario rather than the IR. However, incorporating IRs could transfer the management of the experimental procedure from the scenario to RISE.

### 5.3 Comparison with other systems

As explained in Section 4, RISE is a feature-rich architecture for HRI studies. To differentiate RISE from other systems that also aim to support HRI studies, we emphasize its distinct features that we consider to be particularly important for interdisciplinary HRI research. The system requirements of RISE (Section 3) are compared to those of other systems in terms of interdisciplinarity and reproducibility. Therefore, we define the following key attributes for the five requirements (configurability, introspection, robot control, extensibility, and robot independence): *Configurability* describes the possibility to create different configurations that can be reused fully or partly for other studies. For introspection, the previously described systems (Section 1) list various GUIs (*GUI: introspection*). In addition, a *visual programming* interface simplifies the configuration. For conducting HRI studies, the robot control can allow for full *autonomy* and fully controlled execution (*WoZ*). Again, a GUI would simplify controlling the robot (*GUI: controlling*). *Robot independence* enables the execution of the interaction study with different robotic platforms, which is a key aspect of conceptual reproducibility. The use of common *middleware* allows easy expansion for the purpose of including new robotic platforms or replacing individual processing modules. It is also a good indication of simple *extensibility* of the programming interface for using external features and sensors.

Table 3 provides an overview of the architectures discussed in Section 2. By highlighting the key attributes listed there, we can illustrate the advantages and capabilities of RISE in the context of HRI research. With the configurability of CRAs and IRs, RISE offers an easy way to define robot behavior (Section 4.2).

**TABLE 3:** Architecture comparison with a focus on key attributes for interdisciplinarity and reproducibility.

	Interdisciplinarity				Reproducibility				
	Configurability	GUI: controlling	Visual programming	GUI: introspection	Robot-independent	Extensibility	Middleware	Wizard of Oz	Autonomy
RISE	✓	✓	(✓)	✓	✓	✓	ROS	✓	✓
Choregraphe	✓	✓	✓	✓	✗	(✓)	✗	✓	✓
OpenDial	✓	✗	✗	✓	(✓)	(✓)	**?**	✗	✓
Bonsai	✓	✗	✗	✗	**?**	✓	**?**	✗	✓
WoZ4U	✓	✓	✗	✗	✗	✗	✗	✓	✗
Polonius	✓	✓	✗	✗	✓	✗	ROS	✓	✗
PaMini	✓	✗	✗	✗	✓	✓	RSB	✗	✓
Interaction Composer	✓	**?**	✓	**?**	✓	✓	**?**	**?**	✓
IrisTK	✓	✗	✗	✗	✗	✓	**?**	✗	✓
InproTK	✓	✗	✗	✗	✓	✓	RSB	✗	✓

For a prior state of the CRAs (Groß et al., 2022), a visual programming component was designed and tested (Schütze et al., 2022). Here, domain experts with no knowledge about robotics were able to understand, change, and design these configurations using the visual programming component. The visual programming component is unavailable in the runtime version of the current RISE version because of package incompatibilities. For future updates, these components should be adjusted and integrated to enhance the usability.

The GUI of RISE reveals the inner states of the system and can explain the robot behavior (Section 4.4). Researchers can either control the robot behavior themselves or execute predesigned IRs to automate its behavior. In order to enhance the automation capabilities, RISE offers the possibility to connect external sensors and features and to respond to events occurring in different scenarios, e.g., a touchscreen. Since it is released as an open source, RISE enables the expansion of its capabilities by integrating additional robots and their distinct modalities. RISE offers features of extensibility because of its external nodes and its independence from specific robots. Due to the language independence of the ROS framework, it allows for the implementation of libraries in various programming languages^25^.

In summary, when considering the essential attributes in terms of functionality, most of the different systems are either designed for WoZ or for autonomous interaction. The two systems, OpenDial and Choregraphe, show the greatest similarity to our architecture in terms of functional scope, each of which specializes in certain aspects of the functions, but neither of the two encompasses all the essential attributes. OpenDial offers interesting dialog and speech processing capabilities but is limited to these features. Choregraphe is still a frequently utilized software program for both WoZ and autonomous studies. However, its limitation to NAO and Pepper, as well as the restriction of extensibility because of its outdated SDK, hinders the utilization of its full potential. RISE is unique in that it allows for the control of various robot platforms and does not limit itself to a single specialized application. RISE can, for instance, function as a basic dialog manager, but it is also proficient at supervising and managing a dialog manager in more complex scenarios.

## 6 Discussion

In this article, the concept and architecture for a system were presented, which were intended to simplify the collaborative work of interdisciplinary teams in the field of HRI. By employing a reusable and expandable architecture, the proposed system aims to provide experts with a distinct advantage in developing high-quality HRI scenarios.

Through a proof-of-concept, we showcased that our system is not just a theoretical construct but that it also offers practical advantages. By reviewing past experiments (Section 5.2) that employed real-world study designs and research inquiries, we demonstrated the potential advantages of integrating the proposed system. These analyses shed light on how the adoption of a system would have enriched the results of these studies. Furthermore, by illustrating the extensibility and ability of the system to add new robots and new modalities, we demonstrate the scalability of the system for meeting future requirements in the field of HRI.

For this purpose, the development of RISE was described in this article. RISE was developed to support researchers in creating human–robot scenarios and to provide scientists with options to simplify the implementation of HRI scenarios. With RISE, static behaviors of robots can be configured easily and can be executed by CRAs (Section 4.2.1). This includes the creation of simple or multimodal sequences of actions, such as speech and gestures, which the robot performs during interactions with humans. In addition, it is also possible to freely define configurable robot behavior reactions, based on different signals from the environment, as IRs (Section 4.2.2). RISE can store information from the environment or about the context in a WM (Section 4.2.3). This enables RISE to make all information accessible to the other components in the environment via the ROS. To enhance the transparency of HRI scenarios and the associated robot behavior, a GUI was implemented to make the underlying backend of the robot more accessible and understandable (Section 4.4).

These basic structures of RISE, among other things, simplify the work on HRI studies for interdisciplinary teams. The implementation of scenarios and applications in robots represents a major challenge in HRI. Interdisciplinary teams, with different stakeholders (Glas et al., 2012), face the task of integrating diverse thoughts and concepts from various disciplines. Translating these ideas into tangible robot actions is not always straightforward. To counteract this issue, an intensive exchange between the disciplines is needed. However, since robot behavior is often not transparent enough, researchers’ understanding of a robot’s capabilities is not always identical. The implementation of a GUI in RISE addresses this issue by providing a transparent system that facilitates the exchange of internal states and enables the expression of different reactive behaviors on behalf of the robot in diverse situations. This approach helps mitigate problems of collaboration and enhances the understandability of the system (Colin et al., 2022) for three stakeholder roles (Figure 1) that are foreseen in our system: (1) the researcher, who conducts, monitors, and configures the study, (2) the developer, who technically customizes RISE for adaptation to specific circumstances, and (3) the participant, who actively engages in the interaction with the robot.

Ensuring reproducibility in HRI studies is a crucial concern in order to maintain consistent robot behavior across various setups in different locations (Gunes et al., 2022). In addition, automated construction processes for HRI experiments are employed to enable the reproducibility of study setups (Lier et al., 2014)^26^. Controlling the behavior of robots and programming them poses an additional challenge, typically requiring the application of programming languages and more advanced functions on the robot’s side. Through the abstraction of these functions, RISE provides an interface that caters to all stakeholders, including those without specific programming expertise. RISE allows researchers to design robot behaviors in the XML format (Section 4.2.1) and execute them via the GUI. This not only promotes a more balanced distribution of tasks within a team but also facilitates the understanding of HRI by individuals from various disciplines. Enhanced clarity of functions fosters improved collaboration and facilitates the exchange of actual robot functions, resulting in more effective teamwork (Ylikoski and Aydinonat, 2014). This also empowers experts to focus on specialized features within HRI, as tasks can be delegated and shared among team members. In addition, RISE fosters conceptual reproducibility due to its robot independence.

RISE has so far been examined mostly in a theoretical sense, focusing on its potential to enhance past and future studies in HRI. During a first proof-of-concept study (Groß et al., 2022), we demonstrated that a previous version of RISE exhibited good system usability. The study primarily emphasized the configurability of the system structures and the provision of an intuitive user interface. A comprehensive evaluation of the system itself, involving real interdisciplinary projects and perspectives from different disciplines, is still pending. This evaluation is crucial for the identification of limitations, the refinement of the system, and the ensuring of its effectiveness in practical applications. The challenge lies in achieving a high level of abstraction for robot actions while ensuring the simplicity of the abstraction language without reducing the scope of functionality. By supporting configurations in XML, RISE offers a significant simplification in the design process of robot behaviors. However, the defined syntax of this description language still entails difficulties (Groß et al., 2022). Schütze et al. (2022) focused on the design process of robot behaviors for an alpha version of RISE to reduce the effort of creating behaviors via XML. This work shows that a visual programming component contributes to improving the usability of the system significantly for experts as well as non-experts. To further simplify the design process of configuring HRI scenarios, a visual programming component within RISE will be included in future work.

However, through ongoing system evaluations, our future work aims to enhance the system incrementally, focusing on optimizing its usability and the overall user experience. These ongoing improvements are aimed at enhancing the system to the point where even users without scientific expertise can possibly utilize it in their daily lives. This objective requires the inclusion of additional robots into the system, making them readily selectable by default. By achieving this, new use cases can be explored, potentially leading to the widespread adoption of RISE in various settings, including households.

This work introduces RISE, an open-source architecture designed to foster interdisciplinarity and promote reusability in HRI studies. RISE provides a platform where interdisciplinary teams can collaborate, design, and configure HRI content, facilitating effective communication and collaboration among team members from diverse disciplines working on shared robotic content. To enable the reusability of HRI studies, RISE is introduced as an architecture that can effectively facilitate the mapping of robot-independent, autonomous, and semi-controlled studies. Moreover, the connectivity of the architecture with external components ensures scalability, setting a strong foundation for future advancements in the field of robotics.

## Data Availability

The open-source code of RISE and configurations of the project, described in this article, are available in the repository under the MIT license: https://gitlab.ub.uni-bielefeld.de/rise-projects.

## Footnotes

1 A collaborative research center is a special funding format that targets interdisciplinary research with a joint research hypothesis to which all projects subscribe. Collaboration and joint contribution to the development of underlying theoretical concepts are core ideas behind a CRC. In a CRC, generally, between 15 and 25 principal investigators work together with about 25–30 early-career researchers from different disciplines.

2 TRR318 Constructing Explainability (2021) https://trr318.uni-paderborn.de/en/ [Accessed 21 June 2023].

3 PaMini (2017) https://mvnrepository.com/artifact/de.unibi.agai/PaMini [Accessed 22 June 2023].

4 IrisTK (2012) http://www.iristk.net/ [Accessed 19 June 2023].

5 InproTK (2012) https://www.timobaumann.de/work//Main/InproTK [Accessed 19 June 2023].

6 OpenDial (2016) http://opendial-toolkit.net/ [Accessed 19 June 2023].

7 BonSAI (2011) https://opensource.cit-ec.de/projects/bonsai/ [Accessed 21 June 2023].

8 Choregraphe (2009) http://doc.aldebaran.com/2-5/software/choregraphe/index.html [Accessed 21 June 2023].

9 Polonius (2011) https://wiki.ros.org/polonius/Tutorials/WritingaPoloniusScript [Accessed 21 June 2023].

10 WoZ4U (2021) https://github.com/frietz58/WoZ4U [Accessed 21 June 2023].

11 RISE (2023) https://gitlab.ub.uni-bielefeld.de/rise-projects/application/rise-core [Accessed 21 June 2023].

12 RISE-Documentation https://rise-projects.pages.ub.uni-bielefeld.de/rise-documentation/contents.html [Accessed 21 June 2023].

13 Unity3D https://unity.com [Accessed 21 March 2023].

14 ROS-ActionLib http://wiki.ros.org/actionlib [Accessed 21 June 2023].

15 LogicExpressionParser https://github.com/Bunny83/LSystem [Accessed 21 June 2023].

16 RViz http://wiki.ros.org/rviz [Accessed 04 October 2023].

17
https://gitlab.ub.uni-bielefeld.de/rise-projects/application/rise-configurations/-/tree/main/Tutorials/RobotInitiatedGreeting [Accessed 13 October 2023].

18 Getting Started with RISE https://rise-projects.pages.ub.uni-bielefeld.de/rise-documentation/gettingStarted.html [Accessed 08 September 2023].

19 RISE-Tutorials https://rise-projects.pages.ub.uni-bielefeld.de/rise-documentation/tutorialOverview.html [Accessed 08 September 2023].

20 See Alharbi et al. (2021) for an overview of ASR tools.

21 ASR2ChatGPT https://gitlab.ub.uni-bielefeld.de/rise-projects/feature-nodes/asr2chatgpt [Accessed 20 August 2023].

22 Rosbag http://wiki.ros.org/rosbag [Accessed 21 June 2023].

23 RISE-Configurations, LabLinking https://gitlab.ub.uni-bielefeld.de/rise-projects/application/rise-configurations/-/tree/main/Tutorials/StudyLabLinking [Accessed 13 September 2023].

24 RISE-Configurations, Scaffolding Negation https://gitlab.ub.uni-bielefeld.de/rise-projects/application/rise-configurations/-/tree/main/Tutorials/StudyNegationFloka [Accessed 13 September 2023].

25 Introduction to ROS http://wiki.ros.org/ROS/Introduction [Accessed 21 June 2023].

26 RISE is integrated into the CITK https://gitlab.ub.uni-bielefeld.de/rdtk/citk
